# Genomic Evidence of In-Flight SARS-CoV-2 Transmission, India to Australia, April 2021

**DOI:** 10.3201/eid2807.212466

**Published:** 2022-07

**Authors:** Freya Hogarth, Pasqualina Coffey, Laura Goddard, Sarah Lewis, Shereen Labib, Mathilda Wilmot, Patiyan Andersson, Norelle Sherry, Torsten Seemann, Benjamin P. Howden, Kevin Freeman, Robert Baird, Ian Hosegood, Kathleen McDermott, Nick Walsh, Ben Polkinghorne, Catherine Marshall, Jane Davies, Vicki Krause, Ella M. Meumann

**Affiliations:** Australian Government Department of Health, Canberra, Australian Capital Territory, Australia (F. Hogarth);; The Australian National University, Canberra (F. Hogarth, B. Polkinghorne);; Centre for Disease Control, Darwin, Northern Territory, Australia (P. Coffey, L. Goddard, S. Lewis, S. Labib, V. Krause);; The University of Melbourne at The Peter Doherty Institute for Infection and Immunity, Melbourne, Victoria, Australia (M. Wilmot, P. Andersson, N. Sherry, T. Seemann, B.P. Howden);; Royal Darwin Hospital, Darwin (K. Freeman, R. Baird, C. Marshall, J. Davies, E.M. Meumann);; Qantas Airways Limited, Mascot, New South Wales, Australia (I. Hosegood);; National Critical Care and Trauma Response Centre, Darwin (K. McDermott, N. Walsh);; Menzies School of Health Research and Charles Darwin University, Darwin (J. Davies, E.M. Meumann)

**Keywords:** COVID-19, 2019 novel coronavirus disease, coronavirus disease, severe acute respiratory syndrome coronavirus 2, SARS-CoV-2, viruses, respiratory infections, zoonoses, quarantine, covid-19, genome viral, in-flight transmission, genomics, epidemiology, airplane, India, Australia

## Abstract

Epidemiologic and genomic investigation of SARS-CoV-2 infections associated with 2 repatriation flights from India to Australia in April 2021 indicated that 4 passengers transmitted SARS-CoV-2 to >11 other passengers. Results suggest transmission despite mandatory mask use and predeparture testing. For subsequent flights, predeparture quarantine and expanded predeparture testing were implemented.

During the first epidemic wave of SARS-CoV-2, Australia closed its borders; during March 28, 2020–November 1, 2021, international arriving passengers were required to undergo mandatory supervised quarantine (*1*). This initial response contributed to the end of the first pandemic wave in June 2020 and resulted in periods of COVID-19 control throughout the country (*2*). 

Beginning October 23, 2020, a quarantine facility in Darwin, Northern Territory, Australia, received persons who arrived via government-assisted repatriation flights. On April 15 and 17, 2021, two repatriation flights (flights 1 and 2) carrying passengers from 2 regions of India experiencing major COVID-19 outbreaks landed in Darwin. The percentages of passengers positive for COVID-19 were substantially greater for these 2 flights (24/164 [15%] and 23/181 [13%]) than for all previous repatriation flights to Darwin (225/9,651 [2%] during October 2020–April 2021).

In the 48 hours before flying, all passengers on the 2 flights had tested negative for SARS-CoV-2 by quantitative reverse transcription PCR (qRT-PCR). All passengers except infants and children were required to wear masks (*3*). COVID-19 vaccination coverage among passengers was low; 24/345 (7%) passengers had received >1 dose, and only 14 had received 2 doses of the same vaccine >14 days apart. At arrival, passengers entered quarantine, where they were tested for SARS-CoV-2 by qRT-PCR on days 0, 7, and 12, in addition to testing if symptomatic (Appendix 1).

Of the 47 passengers with positive results, 21 tested positive at arrival (arrival case-patients) and 26 tested positive >1 day after arriving in quarantine (quarantine case-patients) (Appendix 1 Figures 1, 2). Of the 21 arrival case-patients (Table), 18 were asymptomatic. qRT-PCR cycle threshold values were available for 18/21 (86%) arrival case-patients; median was 15.2 (range 8.4–34.1) cycles. For quarantine case-patients, median time of symptom onset was 5 (range 0–8) days after arrival, and the median number of days from arrival to a positive test result was 4 (range 1–7) days.

**Table:** Detailed information of case-patients belonging to SARS-CoV-2 genomic clusters detected after 2 flights from India to Darwin, Northern Territory, Australia, on April 15 and April 17, 2021*

Cluster and case-patient	Age group, y/sex	Family group	Virus Pango lineage	Cycle threshold	Symptom onset date	Date tested positive	Vaccinated	Seat no.
1								
A	30–39/M	None	B.1.617.2	14.3	Asymptomatic	Apr 15	N	56B
B	40–49/M	I	B.1.617.2	15.6	Asymptomatic	Apr 15	N	43D
C	20–29/F	I	B.1.617.2	11.6	Apr 20	Apr 20	N	43E
D	1–5/F	I	B.1.617.2	11.6	Asymptomatic	Apr 20	N	43F
E	<1/M	I	B.1.617.2	12.2	Asymptomatic	Apr 20	N	43D
F	30–39/M	II	B.1.617.2	22.6	Apr 20	Apr 20	N	43K
G	10–19/F	II	B.1.617.2	18	Apr 20	Apr 20	N	43H
H	1–5/M	II	B.1.617.2	26.5	Asymptomatic	Apr 20	N	43J
I	<1/F	II	B.1.617.2	19	Asymptomatic	Apr 20	N	43K
2								
J	20–29/F	None	B.1.617.1	12.4	Apr 16	Apr 15	N	42A
K	50–59/M	None	B.1.617.1	16.6	Apr 17	Apr 20	N	51H
L	1–5/M	III	B.1.617.1	22	Asymptomatic	Apr 22	N	42B
M	1–5/M	III	B.1.617.1	18.1	Apr 22	Apr 22	N	43B
N	30–39/F	III	B.1.617.1	20	Asymptomatic	Apr 22	N	43B
3								
O	50–59/F	IV	B.1.617.2	14.9	Asymptomatic	Apr 15	Y	3E
P	60–69/M	IV	B.1.617.2	14.9	Apr 15	Apr 16	Y	3F
Q	10–19/F	None	B.1.617.2	11.4	Asymptomatic	Apr 17	N	4E
4								
R	50–59/M	V	B.1.617.2	14.9	Asymptomatic	Apr 22	N	55A
S	60–69/F	V	B.1.617.2	14.9	Apr 22	Apr 23	N	55B
5								
T	10–19/M		B.1.617.2	11.7	Apr 17	Apr 18	N	48C
U	30–39/M	VI	B.1.617.2	16.1	Asymptomatic	Apr 24	N	48B
V	30–39/F	VI	B.1.617.2	12.8	Not available	Apr 24	N	48A
W	1–5/M		B.1.617.2	24.5	Asymptomatic	Apr 24	N	48J
6								
X	30–39/M	VII	B.1.1.7	10.9	Asymptomatic	Apr 17	N	43F
Y	40–49/M	VII	B.1.1.7	13.1	Asymptomatic	Apr 17	N	43E

Among 41 (87%) of 47 SARS-CoV-2 genome sequences generated from case-patients on flights and 1 and 2, variant types were Delta (B.1.617.2) for 27 (57%), Kappa (B.1.617.1) for 10 (21%), Alpha (B.1.1.7) for 3 (6%), and A.23.1 sublineage for 1 (2%). Of 41 sequences, 25 (59%) belonged to 1 of 6 genomic clusters (Table; Figure; Appendix 1 Figure 3).

**Figure:**
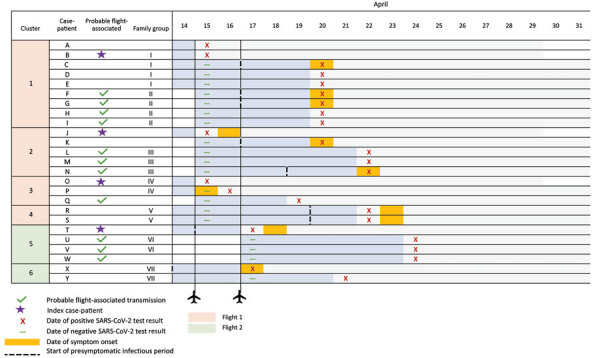
Schematic showing genomic clusters and in-flight transmission of SARS-CoV-2 on 2 flights from India to Australia, April 2021.

To determine whether infections were likely to have been acquired during flight, we analyzed case interviews, flight manifests, and genomic sequencing. Of the 21 arrival case-patients, 4 (19%) (identified as B, J, O, and T) on both flights were likely to have transmitted SARS-CoV-2 to >11 other passengers (F–I, L–N, Q, and U–W) who had sequences that belonged to the same SARS-CoV-2 genomic clusters, who did not belong to the same family group of an arrival case-patient, and who had been seated within 2 rows of an arrival case-patient. Using this information, we calculated secondary attack rates of 6% (8/143) for flight 1 and 2% (3/168) for flight 2. Five case-patients (C–E, P, and Y) with genomically linked virus belonged to arrival case family groups for which transmission possibly occurred before, during, or after the flight. One case-patient (K) with virus belonging to a genomic cluster was seated >2 rows from an arrival case-patient with genomically linked virus. Virus from 2 quarantine case-patients (R and S) genomically linked them to each other but not to an arrival case-patient (Table; Figure; Appendix 1). Only 5 quarantine case-patients from the flights had sequences that did not belong to a SARS-CoV-2 genomic cluster (Appendix 1 Figures 1, 2). Genomics refuted transmission to 6 quarantine case-patients seated within 2 rows of an arrival case-patient, linking 3 to a different cluster.

Soon after the 2 repatriation flights reported here, other repatriation flights from India were suspended, but flights resumed on May 15, 2021, when mandatory 72-hour preflight quarantine of passengers within India was instituted and testing of passengers was expanded to include rapid antigen testing on entry to preflight quarantine, qRT-PCR testing 48 hours before departure, and rapid antigen testing on the day of departure (*4*). During May 15–October 14, 2021, SARS-CoV-2 test results were positive for 13 (0.29%) of 4,543 passengers on repatriation flights from India and 30 (0.28%) of 10,679 passengers on repatriation flights to Darwin. Probable contributors to reduced repatriation cases were increasing vaccination rates and abatement of the Delta wave in India and globally (*5*).

At the time of this study, COVID-19 vaccination rates in Australia were low, most jurisdictions had little or no community transmission of SARS-CoV-2, and quarantine was key to reducing international incursions. We could not exclude transmission in the departure lounge and during boarding; however, spatial proximity of case-patients who did not belong to the same family groups but had genomically linked virus supported in-flight transmission. Previous studies that reported in-flight transmission of SARS-CoV-2 (*6*–*10*) did not include preflight testing, whereas our study included complete preflight and postflight testing and genomic sequencing. In conclusion, our investigation revealed evidence of flight-associated SARS-CoV-2 transmission on 2 repatriation flights from India to Australia during the Delta variant wave in April 2021.

Appendix 1 Supplemental methods and results for study of genomic evidence of in-flight SARS-CoV-2 transmission, India to Australia, April 2021 (Appendix 2).

Appendix 2 Virus samples used in study of genomic evidence of in-flight SARS-CoV-2 transmission, India to Australia, April 2021.
