# Supplementary material for: Genomic Evidence of In-Flight SARS-CoV-2 Transmission, India to Australia, April 2021
Source: Emerg Infect Dis. 2022 Jul;28(7):1527–30. doi: 10.3201/eid2807.212466 (PMC9239893; doi:10.3201/eid2807.212466)
Supplement: Appendix 1 — Supplemental methods and results for study of genomic evidence of in-flight SARS-CoV-2 transmission, India to Australia, April 2021 (Appendix 2). [file 21-2466-Techapp-s1.pdf]

Article DOI: https://doi.org/10.3201/eid2807.212466

Genomic Evidence of In-Flight SARS-CoV-2 Transmission, India to Australia, April 2021

Appendix 1

Management of the flights

On arrival to Darwin, passengers from flight 1 and flight 2 arrived at a separate air terminal then travelled by bus by seating cohorts to the Howard Springs International Quarantine Facility (HSIQF). Passengers were seated at least 1.5 meters apart and masks were mandatory for persons ≥12 years old unless there was a medical contraindication (*1*). At the HSIQF passengers were physically separated into family groups in self-contained units that were well-ventilated with separate, non-communal bathroom facilities.

Individuals with a positive result on routine testing were moved to a separate zone within the facility for COVID-19 management. Close contacts of COVID-19 cases were moved to a second separate zone and required to quarantine for at least 14 days from their last exposure to the case and were monitored daily for fever and respiratory symptoms (*2*). On April 16, 2021, enhanced safety measures including the requirement for staff working with cases or close contacts to be fully vaccinated, wear full personal protective equipment, and undergo daily testing for SARS-CoV-2 by bilateral nasal swab rapid antigen testing in addition to daily saliva PCR confirmation. There were no instances of transmission between family groups or to staff within the HSIQF during this period.

SARS-CoV-2 testing, genomic sequencing, and bioinformatics analysis

Testing for SARS-CoV-2 was performed on swabs collected from the oropharynx and bilateral deep nasal passages, using the RT-qPCR assay from AusDiagnostics (Australia) with primers for the ORF1a and ORF8 genes. RT-qPCR cycle threshold (Ct) values reported in this manuscript are for the ORF8 target.

Genomic sequencing and consensus sequence generation were undertaken at the Microbiological Diagnostic Unit Public Health Laboratory at the Doherty Institute,

Melbourne as described by Lane et al (*3*). In brief, tiled amplicons were generated using the ARTIC version 3 primers (https://github.com/artic-network/artic-ncov2019/tree/master/primer_schemes/nCoV-2019), libraries were prepared using NexteraXT, and sequencing was undertaken on the NextSeq500/550 or iSeq100 (Illumina) using 150bp paired-end reads. Reads were aligned to the Wuhan-Hu-1 reference (Genbank MN908947.3) to generate consensus sequences, which were uploaded to GISAID (https://www.gisaid.org; Appendix 2 Table 1, https://wwwnc.cdc.gov/EID/article/28/7/21-2466-App2.pdf). SARS-CoV-2 lineages were assigned using Pangolin v3.1.5 (*4*,*5*). Genomes belonging to lineages B.1.617.2 (Delta variant) and B.1.617.1 (Kappa variant) were included in a phylogenetic analysis with 300 publicly available SARS-CoV-2 genomes for context. To select context genomes, all 3,162 B.1.617 sequences from India between April 1, 2021, and April 15, 2021 with ≤5% missing or ambiguous base calls were downloaded from GISAID (https://www.gisaid.org) on September 13, 2021, and 300 of these were randomly selected for inclusion (Appendix 2 Table 2). Genomes were aligned to the Wuhan-Hu-1 reference genome using MAFFT v7.464.11 (*6*), and problematic sites (https://github.com/W-L/ProblematicSites_SARS-CoV2#human-friendly-version-of-the-vcf-file; last updated July 28, 2021) were masked from the alignment. Phylogenetic analysis was undertaken using IQ-TREE v1.6.12 (*7*) using a generalised time reversible model with 4 gamma categories and 1,000 ultrafast bootstrap replicates. The phylogenetic tree was annotated using the ggtree package in R v4.0.2. Genomic clusters from the flights were identified by visualisation of the phylogenetic tree.

Case definitions

Unless a negative RT-qPCR result indicated otherwise, we assumed the pre-symptomatic infectious period to be 1–3 days (*2*,*8*,*9*), and the incubation period to range from 1–14 days (*2*,) (C. Daley, unpub. data, https://www.medrxiv.org/content/10.1101/2020.12.23.20248790v1) *10–13*).

We defined arrival cases as passengers on flight 1 and flight 2 landing in Darwin on April 15 and 17 2021 respectively who tested positive to SARS-CoV-2 by RT-qPCR on day 0 of their quarantine period. Quarantine cases were defined as passengers who tested positive to SARS-CoV-2 by RT-qPCR ≥ 1 day after arriving in quarantine. Their infection was determined to result from probable flight-associated transmission if they were seated inside the 2x2 area of an arrival case (that is, within two rows either side of the arrival case); they

were not a travel companion of an arrival case; they returned a SARS-CoV-2 virus genome sequence separated by ≤2 single nucleotide polymorphisms to that of an arrival case in the 2x2 area; and their SARS-CoV-2 genome was located on the same distal clade in the phylogenetic tree as the arrival case in the 2x2 area.

Detail of the clusters

Flight 1

Cluster 1

Cluster 1 included 9 cases with B.1.617.2 (Delta variant) infection (Figure 1; Figure 3). Two arrival cases (arrival cases A and B) belonging to this cluster were asymptomatic and had Ct values of 14.3 and 15.6 cycles. No plausible epidemiologic link prior to the flight was identified between the two arrival cases. Arrival case B, seated in row 43, was within the same 2x2 area as 7 individuals who were subsequently diagnosed with COVID-19 while in quarantine; these included 3 of their own family members (quarantine cases C, D, and E; family group I) and 4 members of a separate family (quarantine cases F, G, H, and I; family group II), all of whom were seated in row 43 and tested positive for SARS-CoV-2 on day 5 of quarantine. Members of family group I may have been infected before, during, or after the flight, while family group II who were travelling in the same row were attributed to probable flight-associated transmission.

Cluster 2

Cluster 2 included 5 cases with B.617.1 (Kappa variant) infection (Figure 1). One case (arrival case J) had a Ct value of 12.4 cycles and developed COVID-19 symptoms on day 1. This case was seated in row 42. Three members of a different family group (quarantine cases L, M, and N; all belonging to family group III) seated in row 43 subsequently tested positive to SARS-CoV-2 on the routine day 7 test. The infections of these three cases were determined to result from probable flight-associated transmission. A further quarantine case (quarantine case K) diagnosed on day 5 of quarantine was seated separately in row 51 and had no known epidemiologic links to the other cases in the cluster.

Cluster 3

Cluster 3 included 3 cases belonging to B.1.617.2 (Delta variant) (Figure 1; Figure 3). One case (arrival case O) seated in row 3 was asymptomatic and had a Ct value of 14.9 cycles. Their partner (quarantine case P; family group IV) had a negative arrival test but

tested positive for SARS-CoV-2 on day 1 of quarantine. A third case (quarantine case Q) seated in row 4, not travelling with the other two cases, tested positive for SARS-CoV-2 on day 2. The infection of this case was determined to result from probable flight-associated transmission.

Cluster 4

Cluster 4 included 2 cases belonging to B.1.617.2 (Delta variant) (Figure 2; Figure 3). The two cases (quarantine case R and S) seated in row 55 belonged to the same family group (family group V) and both tested positive to SARS-CoV-2 on the routine day 7 test.

Flight 2

Cluster 5

Cluster 5 included four cases belonging to B.1.617.2 (Delta variant) (Figure 2; Figure 3). One case (arrival case T) seated in row 48 had a Ct value of 12.8 cycles and developed symptoms on day 1. Three further cases (quarantine cases U, V, and W) in row 48 belonging to two different travelling groups (including family group VI) tested positive for SARS-CoV-2 on routine day 7 testing. The infections of these three cases were determined to result from probable flight-associated transmission.

Cluster 6

Cluster 6 included two cases belonging to B.1.1.7 (Alpha variant) (Figure 2). One case (arrival case X) developed symptoms on day 0 and had a Ct value of 11.7 cycles (Figure 2). A member of their family (quarantine case Y; family group VII) subsequently tested positive for SARS-CoV-2 on day 4.

References

1. Australian Government Department of Health. Coronavirus (COVID-19) advice for international travellers [cited 2021 May 15]. https://www.health.gov.au/news/health-alerts/novel-coronavirus-2019-ncov-health-alert/coronavirus-covid-19-travel-and-restrictions/coronavirus-covid-19-advice-for-international-travellers

2. Australian Government Department of Health. Coronavirus disease 2019 (COVID-19): CDNA National Guidelines for Public Health Units [cited 2021 April 13]. https://www1.health.gov.au/internet/main/publishing.nsf/Content/cdna-song-novel-coronavirus.htm

3. Lane CR, Sherry NL, Porter AF, Duchene S, Horan K, Andersson P, et al. Genomics-informed responses in the elimination of COVID-19 in Victoria, Australia: an observational, genomic epidemiological study. Lancet Public Health. 2021;6:e547–56. PubMed https://doi.org/10.1016/S2468-2667(21)00133-X

4. O'Toole Á, Scher E, Underwood A, Jackson B, Hill V, McCrone JT, et al. Assignment of epidemiological lineages in an emerging pandemic using the pangolin tool. Virus Evol. 2021;7(2):veab064.

5. Rambaut A, Holmes EC, O'Toole Á, Hill V, McCrone JT, Ruis C, et al. A dynamic nomenclature proposal for SARS-CoV-2 lineages to assist genomic epidemiology. Nat Microbiol. 2020;5:1403–7. PubMed https://doi.org/10.1038/s41564-020-0770-5

6. Katoh K, Misawa K, Kuma K, Miyata T. MAFFT: a novel method for rapid multiple sequence alignment based on fast Fourier transform. Nucleic Acids Res. 2002;30:3059–66. PubMed https://doi.org/10.1093/nar/gkf436

7. Nguyen L-T, Schmidt HA, von Haeseler A, Minh BQ. IQ-TREE: a fast and effective stochastic algorithm for estimating maximum-likelihood phylogenies. Mol Biol Evol. 2015;32:268–74. PubMed https://doi.org/10.1093/molbev/msu300

8. World Health Organization. Transmission of SARS-CoV-2: implications for infection prevention precautions. [cited 2020 Sep 14]. https://www.who.int/news-room/commentaries/detail/transmission-of-sars-cov-2-implications-for-infection-prevention-precautions.

9. Wei WE, Li Z, Chiew CJ, Yong SE, Toh MP, Lee VJ. Presymptomatic Transmission of SARS-CoV-2 - Singapore, January 23-March 16, 2020. MMWR Morb Mortal Wkly Rep. 2020;69:411–5. PubMed https://doi.org/10.15585/mmwr.mm6914e1

10. Elias C, Sekri A, Leblanc P, Cucherat M, Vanhems P. The incubation period of COVID-19: A meta-analysis. Int J Infect Dis. 2021;104:708–10. PubMed https://doi.org/10.1016/j.ijid.2021.01.069

11. Lauer SA, Grantz KH, Bi Q, Jones FK, Zheng Q, Meredith HR, et al. The incubation period of coronavirus disease 2019 (COVID-19) from publicly reported confirmed cases: estimation and application. Ann Intern Med. 2020;172:577–82. PubMed https://doi.org/10.7326/M20-0504

12. Quesada JA, López-Pineda A, Gil-Guillén VF, Arriero-Marín JM, Gutiérrez F, Carratala-Munuera C. Incubation period of COVID-19: A systematic review and meta-analysis. Rev Clin Esp (Barc). 2021;221:109–17. https://doi.org/10.1016/j.rceng.2020.08.002

13. Lv Q, Kong D, He Y, Lu Y, Chen L, Zhao J, et al. A SARS-CoV-2 Delta variant outbreak on airplane: vaccinated air passengers are more protected than unvaccinated. J Travel Med. 2021;28:taab161. PubMed https://doi.org/10.1093/jtm/taab161

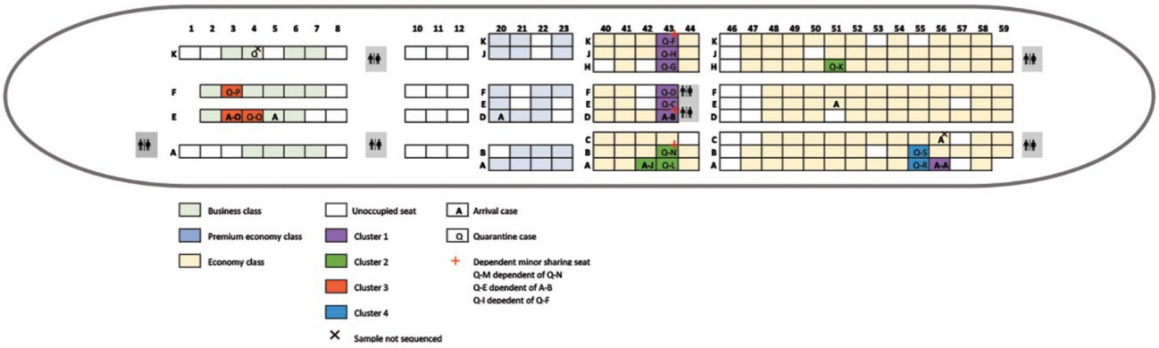

Appendix Figure 1. Seating plan and spatial distribution of arrival and quarantine cases of SARS-CoV-2 aboard flight 1 from India to Darwin, Australia, on April 15, 2021. Occupied seats are shaded. Note that quarantine cases E and I and (cluster 1) and quarantine cases N (cluster 2) were minors sharing a seat with an adult.

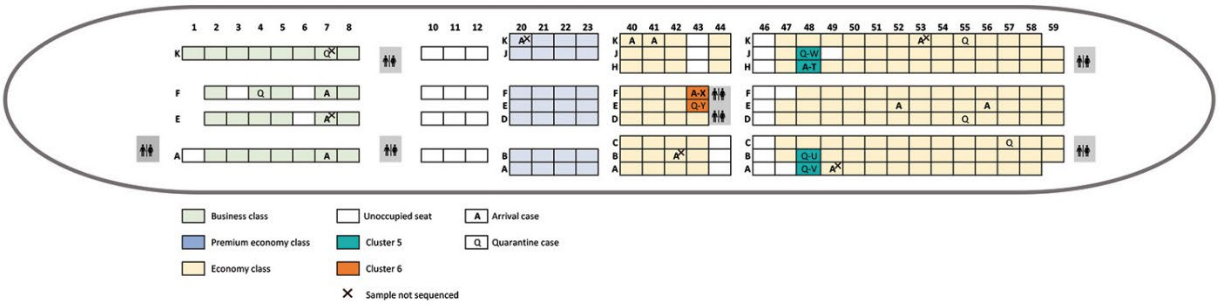

Appendix Figure 2. Seating plan and spatial distribution of arrival and quarantine cases of SARS-CoV-2 aboard flight 2 from India, to Darwin, Australia, on April 17, 2021. Occupied seats are shaded.

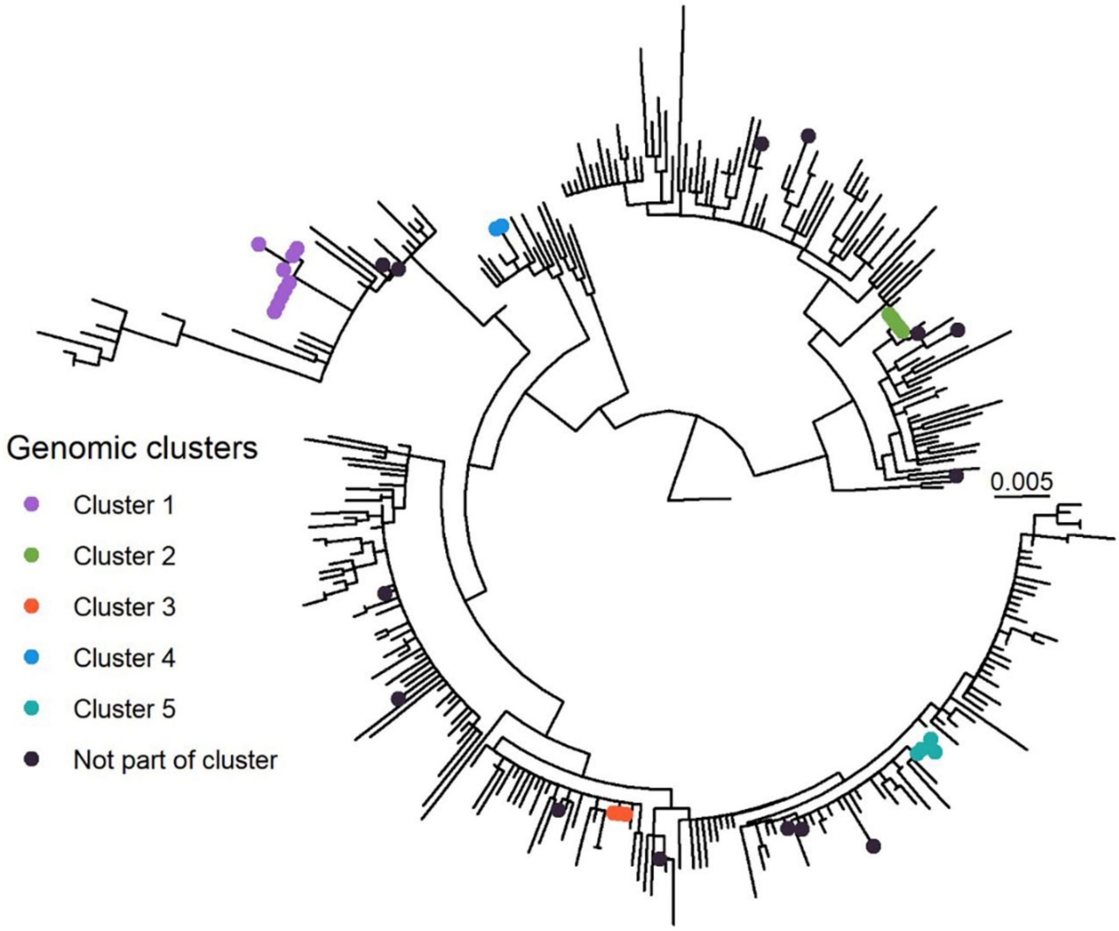

Appendix Figure 3. SARS-CoV-2 B.1.617 maximum likelihood phylogenetic tree including genomes from passengers with COVID-19 from flight 1 from India to Darwin, April 15, 2021, and from flight 2 from India to Darwin, April 17, 2021. The tree was rooted with the MN908947.3 reference genome, and the scale bar indicates substitutions/site.
